# Influenza Resurgence after Relaxation of Public Health and Social Measures, Hong Kong, 2023

**DOI:** 10.3201/eid2912.230937

**Published:** 2023-12

**Authors:** Weijia Xiong, Benjamin J. Cowling, Tim K. Tsang

**Affiliations:** The University of Hong Kong School of Public Health, Pokfulam, Hong Kong, China (W. Xiong, B.J. Cowling, T.K. Tsang);; Hong Kong Science and Technology Park**,** Pak Shek Kok, Hong Kong (B.J. Cowling, T.K. Tsang)

**Keywords:** Influenza, reproductive number, impact of public health and social measures, face mask, viruses, COVID-19, Hong Kong, respiratory infections

## Abstract

Soon after a mask mandate was relaxed (March 1, 2023), the first post–COVID-19 influenza season in Hong Kong lasted 12 weeks. After other preventive measures were accounted for, mask wearing was associated with an estimated 25% reduction in influenza transmission. Influenza resurgence probably resulted from relaxation of mask mandates and other measures.

To control COVID-19, Hong Kong, China, put in place several public health and social measures (PHSMs), including mandatory mask wearing, school closures, hand hygiene, and avoidance of gatherings. In early 2020, those measures also reduced influenza transmission (*1*), and according to laboratory surveillance records, influenza virus did not circulate in the community for 3 years (*2*). From mid-2022 through 2023, PHSMs were progressively relaxed, and on March 1, 2023, the local mask mandate was lifted. We investigated the effects of PHSMs on influenza transmission in Hong Kong.

We collected weekly influenza-like illness consultation rates reported by private general practitioners and the weekly proportion of sentinel respiratory specimens that tested positive for influenza virus in Hong Kong during October 2010–May 2023. We established a proxy for influenza virus activity by multiplying rates of influenza-like illness by the proportion of influenza-positive samples following previous studies (*3*,*4*) (Appendix). We found that weekly influenza activity had decreased to almost zero since March 2020, when PHSMs against COVID-19 began (Figure). Before mandatory on-arrival quarantine of travelers started on September 26, 2022, only sporadic influenza-positive samples were detected by surveillance, all from travelers or children who had recently received live-attenuated influenza vaccine (*5*). After travel restrictions were removed, sporadic influenza detections increased, but overall activity remained low. After mandatory indoor and outdoor mask wearing restrictions were lifted on March 1, 2023, influenza transmission increased substantially; the first influenza season after COVID-19 in Hong Kong started and peaked on April 9, ended on May 25, and lasted for 12 weeks (*6*).

**Figure:**
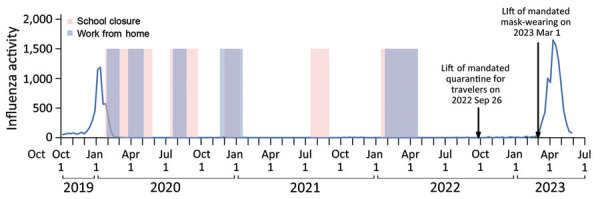
Weekly influenza activity and several preventive measures against COVID-19 in Hong Kong during 2020–2023. The blue line represents the weekly influenza activity, measured by the product of influenza-like illness rates and laboratory detections of influenza.

Because various other PHSMs were implemented concurrently with the mask mandate, resurgence of influenza activity could not be attributed to relaxation of the mask mandate alone. Therefore, we used a previous approach that estimated the time-varying effective reproductive number (R_t_) (*7*) and a multivariable log-linear regression model on R_t_ that could allow for adjustment of other factors affecting influenza transmission, including depletion of susceptible persons, seasonal differences, and meteorologic predictors and preventive measures (Appendix). Because the predominating influenza strain in 2023 was influenza A(H1N1)pdm09, we identified previous influenza A(H1N1)pdm09 epidemics that had occurred during 2010–2020. To construct a preventive score, we used data from cross-sectional telephone surveys among the general adult population in Hong Kong from 2020 to 2023 as a proxy for the intensity of preventive measures, other than mask wearing, against COVID-19 (*1*). The preventive score included the average proportion of persons who avoided visiting crowded places, avoided going to healthcare facilities, avoided touching public objects, or used protective measures when touching public objects, and washed hands immediately after going out. Before 2020, the proportion of those preventive measures was established as baseline. When constructing a preventive score, we compared the Akaike information criterion of 4 combinations of those protective measures. Meteorologic variables provided by the Hong Kong Observatory (http://hko.gov.hk) were temperature, wind speed, and relative and absolute humidity. To quantify the effects of meteorologic variables, we fitted the models to data before the COVID-19 pandemic.

Among the 9 epidemics of 2010–2023, the estimated R_t_ varied from 0.62 to 1.38 (median 1.02) (Appendix Figure 1). The estimated R_t_ showed a decreasing pattern in each season, ranging from ≈1.2 at the beginning of an epidemic period to 0.8 at the end of an epidemic period. After model selection (Appendix), we found that a model of absolute humidity, mask wearing, and preventive score 3 (Table) explained 92% of the observed variance in estimated R_t_ (Appendix Table 1). Changes in absolute humidity (Appendix Figure 2, panel A), the proportion of mask wearing, and preventive score 3 (Appendix Figure 2, panel B) strongly correlated with changes in R_t_. After adjusting for other factors, such as depletion of susceptible persons, between-season effects, and absolute humidity, we found that mask wearing was associated with a 25% (range 1%–43%) reduction in R_t_ and that other preventive measures (combined) were associated with a 77% (range 60%–88%) reduction (Table).

**Table:** Effects of public health and social measures to protect against COVID-19 on R_t_ for influenza, Hong Kong, 2010–2023*

Model†	PHSM description	% Change in R_t_ (95% CI)	ΔAIC‡
Model 1			3.62
Mask		−25 (−43 to −1)	
Preventive score 1	Avoid social gatherings. Wash hands after being outside. Avoid touching or use protective measures with shared objects.	−82 (−91 to −63)	
Model 2			9.55
Mask		−26 (−44 to −2)	
Preventive score 2	Avoid going out as much as possible. Wash hands after being outside. Avoid touching or use protective measures with shared objects.	−80 (−91 to −55)	
Model 3 (main model)			0
Mask		−25 (−43 to −1)	
Preventive score 3	Avoid going to crowded places. Avoid going to healthcare facilities. Avoid touching or use protective measures with shared objects.	−77 (−88 to −60)	
Model 4			
Mask		−24 (−43 to 0)	
Preventive score 4	Avoid going to crowded places. Avoid going to healthcare facilities. Avoid touching or use protective measures with shared objects. Wash hands after being outside.	−81 (−90 to −62)	2.79

*AIC, Akaike information criterion; R_t_, time-varying effective reproductive number. †Models were adjusted for depletion of susceptible persons, between-season effects, and absolute humidity. ‡ΔAIC*_modeli_* = AIC*_modeli_* − AIC*_min_*,AIC*_min_* = *min*(AIC*_modeli_*), *i* = 1,…,4.

We found that that influenza increased after PHSMs were relaxed and influenza transmission increased shortly after the mask mandate was relaxed. Our results are consistent with those of several studies that found that PHSMs against COVID-19 may reduce influenza transmission (*8*) and that mask wearing may have a low to moderate protective effect against influenza virus transmission in the community (*9*,*10*).

A limitation of our analysis was that we used results of survey reports to generate a proxy of intensity of implemented PHSMs over time, which may not be accurate. Also, we used a proxy measure of influenza activity based on surveillance data, and the reliability of our analysis depended on the accuracy of this proxy. In addition, influenza vaccination coverage (Appendix Figure 5) was not included in the model because our model included the effect of vaccination via season-specific intercept. Nevertheless, our study results suggest that the resurgence of influenza after relaxation of PHSMs was most likely affected by the lifting of mask mandate and other PHSMs.

Appendix Additional information for study of influenza resurgence after relaxation of public health and social measures, Hong Kong, 2023.

## References

[1] Cowling BJ, Ali ST, Ng TWY, Tsang TK, Li JCM, Fong MW, et al. Impact assessment of non-pharmaceutical interventions against coronavirus disease 2019 and influenza in Hong Kong: an observational study. Lancet Public Health. 2020;5:e279–88. 10.1016/S2468-2667(20)30090-6 32311320 PMC7164922

[2] Centre for Health Protection, Department of Health, The Goverment of the Hong Kong Special Administrative Region. Flu express [cited 2023 Aug 23]. https://www.chp.gov.hk/files/pdf/fluexpress_week4_2_2_2023_eng.pdf

[3] Goldstein E, Cobey S, Takahashi S, Miller JC, Lipsitch M. Predicting the epidemic sizes of influenza A/H1N1, A/H3N2, and B: a statistical method. PLoS Med. 2011;8:e1001051. 10.1371/journal.pmed.1001051 21750666 PMC3130020

[4] Wu P, Presanis AM, Bond HS, Lau EHY, Fang VJ, Cowling BJ. A joint analysis of influenza-associated hospitalizations and mortality in Hong Kong, 1998-2013. Sci Rep. 2017;7:929. 10.1038/s41598-017-01021-x 28428558 PMC5430505

[5] Mak GCK, Lau SSY, Wong KKY, Lau AWL, Hung DLL. Low prevalence of seasonal influenza viruses in Hong Kong, 2022. Influenza Other Respir Viruses. 2023;17:e13123. 10.1111/irv.13123 36935847 PMC10020920

[6] Centre for Health Protection, Department of Health, The Goverment of the Hong Kong Special Administrative Region. CHP announces end of influenza season [cited 2023 Aug 23]. https://www.info.gov.hk/gia/general/202305/25/P2023052500456.htm

[7] Cori A, Ferguson NM, Fraser C, Cauchemez S. A new framework and software to estimate time-varying reproduction numbers during epidemics. Am J Epidemiol. 2013;178:1505–12. 10.1093/aje/kwt133 24043437 PMC3816335

[8] Fong MW, Gao H, Wong JY, Xiao J, Shiu EYC, Ryu S, et al. Nonpharmaceutical measures for pandemic influenza in nonhealthcare settings—social distancing measures. Emerg Infect Dis. 2020;26:976–84. 10.3201/eid2605.190995 32027585 PMC7181908

[9] Cowling BJ, Chan K-H, Fang VJ, Cheng CK, Fung RO, Wai W, et al. Facemasks and hand hygiene to prevent influenza transmission in households: a cluster randomized trial. Ann Intern Med. 2009;151:437–46. 10.7326/0003-4819-151-7-200910060-00142 19652172

[10] Brienen NC, Timen A, Wallinga J, van Steenbergen JE, Teunis PF. The effect of mask use on the spread of influenza during a pandemic. Risk Anal. 2010;30:1210–8. 10.1111/j.1539-6924.2010.01428.x 20497389 PMC7169241

