# Supplementary material for: Influenza Resurgence after Relaxation of Public Health and Social Measures, Hong Kong, 2023
Source: Emerg Infect Dis. 2023 Dec;29(12):2556–9. doi: 10.3201/eid2912.230937 (PMC10683823; doi:10.3201/eid2912.230937)
Supplement: Appendix — Additional information for study of influenza resurgence after relaxation of public health and social measures, Hong Kong, 2023. [file 23-0937-Techapp-s1.pdf]

Article DOI: https://doi.org/10.3201/eid2912.230937

EID cannot ensure accessibility for supplementary materials supplied by authors. Readers who have difficulty accessing supplementary content should contact the authors for assistance.

Influenza Resurgence after Relaxation of Public Health and Social Measures, Hong Kong, 2023

Appendix

1 Description of the Time Series

1.1 The time series for ILI

We collected the weekly consultation rates of influenza-like illness (ILI) reported by Private Medical Practitioner (PMP) Clinics (chp.gov.hk) and the weekly proportion of sentinel respiratory specimens that tested positive for influenza viruses in Hong Kong from October 2010 through May 2023.

To measure the influenza virus activity, we multiplied the ILI rates with the proportions of influenza-positive specimens together to obtain an influenza proxy (*1*). This influenza proxy shows a stronger correlation with the incidence of influenza virus infections in the community than either influenza-like illness rates or laboratory detection rates alone. We then first multiplied the weekly ILI rates by a constant 70, based on the previous record number of general practitioners in the surveillance. In addition, we divided it by 0.9 as a health seeking(HS) proportion for ILI symptoms in Hong Kong (*2*) and divided it by 0.3 as 30% of influenza cases have ILI symptoms (*3*). To ensure consistency with expected population-level infection rates, we used the constant to scale up the proxy values (*4*,*5*). Finally, we used flexible cubic splines to interpolate daily influenza proxy values from the weekly data.

To identify influenza epidemics, we defined each season's influenza epidemic as a period of at least 12 or more consecutive weeks during which the epidemic baseline was exceeded. The epidemic baseline was determined as 40% quantile of all the non-zero weekly influenza proxy for each influenza season (*6*).

1.2 Time series of meteorological data

We retrieved 10 meteorological predictors provided by Hong Kong Observatory (hko.gov.hk), including pressure, temperature, relative humidity, amount of cloud, rainfall, number of hours of reduced visibility, total bright sunshine, global solar radiation, evaporation, and wind speed. Due to high correlations among these variables, we selected temperature, wind speed, and absolute humidity based on previous literature (*5*,*7*,*8*). We derived the daily mean absolute humidity from the mean relative humidity and mean temperature (*7*,*9*), and then obtained the daily and weekly absolute humidity.

1.3 Time series of preventive measures

We did cross-sectional telephone surveys among the general adult population in Hong Kong from 2020 to 2023 (*10*). The methods and survey instruments used were similar to those used for surveys during the SARS epidemic in 2003 (*11*,*12*) the influenza A H1N1 pandemic in 2009 , and the influenza A H7N9 outbreak in China in 2013 (*13*). Participants were recruited using random-digit dialling of both landline and mobile telephone numbers. Telephone numbers were randomly generated by a computer system. Calls were made during both working and non-working hours by trained interviewers to avoid over-representation of non-working groups. Respondents were required to be at least 18 years old and able to speak Cantonese or English. New respondents were recruited for each survey round. Within each household, an eligible household member with the nearest birthday was invited to participate in the survey, which was not necessarily the person that initially answered the telephone. Survey items included measures of risk perception, attitudes towards COVID-19, and preventive measures taken against contracting COVID-19, including hygiene, face masks, and reduction of social contact. All participants gave verbal informed consent. The prevalence of those preventive measures prior to 2020 was set to be the baseline prevalence.

To proxy the intensity of preventive measures against COVID -19 other than mask wearing, we used data from the survey to construct a preventive score (e.g., the average of proportions of people avoiding visiting crowded places, avoiding touching public objects or using protective measures when touching public objects, and washing hands immediately after going out).

2 Estimation of time-varying effective reproductive number

2.1 Model details

We used the framework in Cori et al (*14*) to estimate the R_t from real data. In brief, it assumes that the distribution of infectiousness through time after infection is independent of calendar time. Transmission then is modelled by using a Poisson process. Denote w_s a probability distribution of the infectiousness profile since infection, therefore the rate for infection at time step t-s generates new infections in time step t is equal to $R_t w_s$, where R_t is the instantaneous reproductive number at t. Also, the incidence at time t is Poisson distributed with mean $R_t \sum_{s=1}^{t} I_{t-s} w_s$.

Denote Y_k the actual (but unobserved) number of new local cases infected on day k. Then, we have:

$$Y_t \sim \text{Poisson}\{R_t \sum_{k=1}^{t-1} Y_k w_{t-k}\}$$

where R_t are the time-varying effective reproductive number at time t respectively.

2.2 Likelihood function

We used the smoothing method as in Cori et al., assuming that the transmissibility is constant over a time period $[t - \tau + 1, t]$, where τ is the smoothing parameter. Hence likelihood at a time period t is

$$P(Y_t, \dots, Y_{t-\tau+1} | Y_1, \dots, Y_{t-\tau}) = \prod_{s=t-\tau+1}^{t} \frac{\left(R_t^\tau \phi(s)\right)^{Y_s} e^{-R_t^\tau \phi(s)}}{Y_s!}$$

where $\phi(t) = \sum_{k=1}^{t-1} Y_k w_{t-k}$. The total likelihood is the product of individual likelihood at each time t in the observed data. The first $\tau - 1$ days were excluded due to τ-day smoothing.

2.3 Priors

We assumed the prior for R_t is Gamma(1,1.5) with mean and standard deviation equal to 1.5.

2.4 Estimation of model parameters

We conducted our analysis in a Bayesian framework and used a Markov chain Monte Carlo (MCMC) algorithm to estimate model parameters. At each MCMC step k, we update the

model parameters θ by using random walk Metropolis-Hastings algorithm (*15*). The step size of the proposal was adjusted to have acceptance rate for 20-30%.

2.5 Assumption on input parameter in data analysis

We use the estimated distribution with mean 2.7 days (*16*) for serial interval. The empirical distribution of reporting delay would be used for a deconvolution approach by Miller et al. (*17*) to obtain the epidemic curve by infection time, which was achieved by using the 'fit_incidence' function in the 'incidental' package in R.

We analyse the epidemic curve up to 31 May 2023, and take $\tau = 14$ in our analysis, to avoid unstable estimates for time-varying reproductive number.

2.6 Inference

After obtaining the epidemic curve by infection time, we use the model in Section 2.1 to estimate Rt. We use a Markov chain Monte Carlo approach to estimate the model parameter, as stated in Section 2.4.

We accounted for the uncertainty of input parameters, including incubation period and infectiousness profile to obtain the final estimates of R_t in addition to model parameter uncertainty as follows:

We followed the bootstrap approach (*18*,*19*) to account for the uncertainty of input parameters, including incubation period, to obtain the final estimates of R_t in addition to uncertainty of model parameters. In each iteration, we use the above deconvolution approach to reconstruct the epidemic curve by infection dates. Then we use above approach to estimate R_t. We presented the mean, 2.5% and 97.5% quantiles for those R_t estimates for each time point across the 200 bootstrap iterations.

3 Construction of Multivariable Regression Models

We used multivariable log-linear regression models to investigate the underlying association between the transmissibility of influenza and different driving factors.

For meteorological factors, we tested different regression forms to investigate the underlying association between the transmissibility of influenza and different plausible driving forces. We compared linear form ($i.e. f_{kt},$ where f_{kt} are the $k-$th drivers), exponential form

$(i.e. \varphi(f_{kt}), where\ \varphi(f_{kt}) = \exp(f_{kt}))$, power form $(i.e. \varphi(f_{kt}) = {f_{kt}}^2)$ of associations across all the meteorological drivers with influenza transmissibility (Table S1).

Following the epidemic model theory and the from aforementioned results, we construct a general multivariable nonlinear regression model described by te Beest et al (*20*). Consider the S_{0j} is the susceptibles (fraction) at the start of i th weeks of j th epidemic and R_0 is the basic reproduction number. Therefore, the instantaneous reproduction number R_{ij} can be written as

$$log(R_{ij}) = log(R_0 S_{0j}) + z_j h_{ij} + \sum_k \beta_k \log(d_{ijk}) + \sum_l \varphi(f_{ijl}) + \epsilon_{ij]}$$

R_{ij} is the time varying instantaneous reproduction number on day i of epidemic j. S_{0j} represents the initial fraction of susceptibles at the start of season j, h_{ij} is the observed cumulative incidence of general practitioner consultations by patients with ILI up to week i−1 of season j, and z_j is a seasonal effect that adjusts h_{ij} to the season-effect. In addition, the season-specific intercept could capture the pre-season influenza vaccine effect. The effect of the driving factors (d_{ijk} or f_{ijl}) during week i for the epidemic j, is determined by the respective coefficients. We treated the parameters $log(R_0 S_{0j})$ and z_j as the nuisance parameters. The coefficient β_k represents the association between R_{ij} and d_{ijk}. $\epsilon_{ij} \sim N(0, \sigma^2)$ is the error term.

We finally define a baseline model based on intrinsic factors (depletion of susceptibles over time and between-season effects) only.

i.e. $log(R_0 S_{0j}) + z_j h_{ij} + \epsilon_{ij}$.

Improved models including other significant factors were then created (Table S2). R-squared (R2) was used to quantify the effects of each factor. Therefore, these ΔR^2 measures (comparing the R-square values of these models) indicate the variance in transmissibility explained by respective drivers.

4 Reference

1. Goldstein E, Cobey S, Takahashi S, Miller JC, Lipsitch M. Predicting the epidemic sizes of influenza A/H1N1, A/H3N2, and B: a statistical method. PLoS Med. 2011;8:e1001051. PubMed https://doi.org/10.1371/journal.pmed.1001051

2. Zhang Q, Feng S, Wong IOL, Ip DKM, Cowling BJ, Lau EHY. A population-based study on healthcare-seeking behaviour of persons with symptoms of respiratory and gastrointestinal-related infections in Hong Kong. BMC Public Health. 2020;20:402. PubMed https://doi.org/10.1186/s12889-020-08555-2

3. Ip DKM, Lau LLH, Chan K-H, Fang VJ, Leung GM, Peiris MJS, et al. The dynamic relationship between clinical symptomatology and viral shedding in naturally acquired seasonal and pandemic influenza virus infections. Clin Infect Dis. 2016;62:431–7. PubMed

4. Wu P, Presanis AM, Bond HS, Lau EHY, Fang VJ, Cowling BJ. A joint analysis of influenza-associated hospitalizations and mortality in Hong Kong, 1998-2013. Sci Rep. 2017;7:929. PubMed https://doi.org/10.1038/s41598-017-01021-x

5. Ali ST, Cowling BJ, Wong JY, Chen D, Shan S, Lau EHY, et al. Influenza seasonality and its environmental driving factors in mainland China and Hong Kong. Sci Total Environ. 2022;818:151724. PubMed https://doi.org/10.1016/j.scitotenv.2021.151724

6. Yang W, Cowling BJ, Lau EH, Shaman J. Forecasting influenza epidemics in Hong Kong. PLOS Comput Biol. 2015;11:e1004383. PubMed https://doi.org/10.1371/journal.pcbi.1004383

7. Shaman J, Kohn M. Absolute humidity modulates influenza survival, transmission, and seasonality. Proc Natl Acad Sci U S A. 2009;106:3243–8. PubMed https://doi.org/10.1073/pnas.0806852106

8. Peci A, Winter A-L, Li Y, Gnaneshan S, Liu J, Mubareka S, et al. Effects of absolute humidity, relative humidity, temperature, and wind speed on influenza activity in Toronto, Ontario, Canada. Appl Environ Microbiol. 2019;85:e02426–18. PubMed https://doi.org/10.1128/AEM.02426-18

9. Wu P, Goldstein E, Ho LM, Yang L, Nishiura H, Wu JT, et al. Excess mortality associated with influenza A and B virus in Hong Kong, 1998-2009. J Infect Dis. 2012;206:1862–71. PubMed https://doi.org/10.1093/infdis/jis628

10. Cowling BJ, Ali ST, Ng TWY, Tsang TK, Li JCM, Fong MW, et al. Impact assessment of non-pharmaceutical interventions against coronavirus disease 2019 and influenza in Hong Kong: an observational study. Lancet Public Health. 2020;5:e279–88. PubMed https://doi.org/10.1016/S2468-2667(20)30090-6

11. Leung GM, Quah S, Ho L-M, Ho S-Y, Hedley AJ, Lee H-P, et al. A tale of two cities: community psychobehavioral surveillance and related impact on outbreak control in Hong Kong and Singapore during the severe acute respiratory syndrome epidemic. Infect Control Hosp Epidemiol. 2004;25:1033–41. PubMed https://doi.org/10.1086/502340

12. Leung GM, Ho L-M, Chan SK, Ho S-Y, Bacon-Shone J, Choy RY, et al. Longitudinal assessment of community psychobehavioral responses during and after the 2003 outbreak of severe acute respiratory syndrome in Hong Kong. Clin Infect Dis. 2005;40:1713–20. PubMed https://doi.org/10.1086/429923

13. Wu P, Fang VJ, Liao Q, Ng DM, Wu JT, Leung GM, et al. Responses to threat of influenza A(H7N9) and support for live poultry markets, Hong Kong, 2013. Emerg Infect Dis. 2014;20:882–6. PubMed https://doi.org/10.3201/eid2005.131859

14. Cori A, Ferguson NM, Fraser C, Cauchemez S. A new framework and software to estimate time-varying reproduction numbers during epidemics. Am J Epidemiol. 2013;178:1505–12. PubMed https://doi.org/10.1093/aje/kwt133

15. Gilks WR, Richardson S, Spiegelhalter D. Markov chain Monte Carlo in practice. London: CRC press; 1995.

16. Tsang TK, Cauchemez S, Perera RA, Freeman G, Fang VJ, Ip DK, et al. Association between antibody titers and protection against influenza virus infection within households. J Infect Dis. 2014;210:684–92. PubMed https://doi.org/10.1093/infdis/jiu186

17. Miller AC, Hannah LA, Futoma J, Foti NJ, Fox EB, D'Amour A, et al. Statistical deconvolution for inference of infection time series. Epidemiology. 2022;33:470–9. PubMed https://doi.org/10.1097/EDE.0000000000001495

18. Salje H, Cummings DAT, Rodriguez-Barraquer I, Katzelnick LC, Lessler J, Klungthong C, et al. Reconstruction of antibody dynamics and infection histories to evaluate dengue risk. Nature. 2018;557:719–23. PubMed https://doi.org/10.1038/s41586-018-0157-4

19. Tsang TK, Wu P, Lau EHY, Cowling BJ. Accounting for imported cases in estimating the time-varying reproductive number of coronavirus disease 2019 in Hong Kong. J Infect Dis. 2021;224:783–7. PubMed https://doi.org/10.1093/infdis/jiab299

20. te Beest DE, van Boven M, Hooiveld M, van den Dool C, Wallinga J. Driving factors of influenza transmission in the Netherlands. Am J Epidemiol. 2013;178:1469–77. PubMed https://doi.org/10.1093/aje/kwt132

Appendix Table 1. AIC values for the model incorporating intrinsic factors and different forms of meteorological drivers to identify the best form of association of R_t and meteorological drivers*

	ΔAIC for associations of influenza and drivers		
**Forms of Association**	**Mean temperature**	**Mean wind speed**	**Mean absolute humidity**
**Linear**	6.89	0.0047	1.39
**Exponential**	3.08	0.0036	0.43
**Power**	0	0	0

$* \Delta AIC_i = AIC_i - AIC_{min}, AIC_{min} = min(AIC_{Linear}, AIC_{Exponential}, AIC_{Power}), i = Linear, Exponential, Power.$

Appendix Table 2. Variance Explained by the Driving Factors of Influenza Transmission in the Hong Kong, $2010-2023$

Driving Factor	Regression Terms M^{*}	$R^{2\dagger}$	$\Delta R^{2\ddagger}$	df	$\boldsymbol{R}_{\text{adj}}^{2}$ §	P value
Depletion of susceptibles	zh_{ij}	0.461	0.461	158	0.458	< 0.001
Between-season effect	$\log\left(R_0 S_{0j}\right)+z_j h_{ij}$	0.899	0.438	142	0.887	< 0.001
Absolute humidity	$\log\left(R_0 S_{0j}\right)+z_j h_{ij}+\beta_1 \varphi(d_{ij1})$¶	0.903	0.004	141	0.891	0.021
Mask	$\log\left(R_0 S_{0j}\right)+z_j h_{ij}+\beta_2 d_{ij2}$	0.902	0.003	141	0.900	0.044
Preventive score 3	$\log\left(R_0 S_{0j}\right)+z_j h_{ij}+\beta_3 d_{ij3}$	0.912	0.013	141	0.901	1.009e-05
Final model	$\log\left(R_0 S_{0j}\right)+z_j h_{ij}+\beta_1 \varphi\left(d_{ij1}\right)$¶ $+\beta_2 d_{ij2}$ $+\beta_3 d_{ij3}$	0.920	0.008	139	0.908	0.001
School holidays	$\log\left(R_0 S_{0j}\right)+z_j h_{ij}+\beta_4 d_{ij4}$	0.902	0.003	141	0.889	0.057
Temperature	$\log\left(R_0 S_{0j}\right)+z_j h_{ij}+\beta_5 \varphi(d_{ij5})$¶	0.909	0.010	141	0.897	0.0001

*All fitted regression models take the form $\log\left(R_{ij}\right)=M+\varepsilon$, and the regression terms M differ for each model.

† R^2 is the variance of the influenza reproduction numbers that is explained by each model.

‡ ΔR^2 is the proportion of the variance explained by a specific driving factor.

§R_{adj}^2 provides a measure of parsimony for each model.

¶ φ is the power form association.

Appendix Table 3. Estimates of the Strength of Driving Factors of Final Model on Influenza Transmission in the Hong Kong, 2010–2023

Driving Factor	Variable	Estimate	95% CI	P value
Between-season intercept	Median $\left[S_{0j}\right]^{*}$	0.833	0.635, 1.085	
Between-season depletion of susceptibles	Median $\left[z_j\right]^{*}$	-0.176	-0.222, -0.153	
Absolute humidity	β_1	-0.051	-0.083, -0.018	0.003
Mask	β_2	-0.288	-0.563, -0.01	0.039
Preventive score 3	β_3	-1.497	-2.086, -0.908	<0.001

$*$ For regression coefficients that were specific for each season, we estimated the range of $R_0 S_{0j}$ to be 0.794-0.993 and the range of z_j to be -1.622 to -0.086 .

Appendix Table 4. Several non-pharmaceutical interventions (NPIs) included in the study

Control measure	Description	Time period	Notes
Mandate quarantine			
Departure location	Compulsory quarantine is required for inbound travellers from specific or all overseas countries/regions.	02/08/2020 – 12/28/2022<br>12/29/2020 – 03/31/2022<br>04/01/2022 – 08/11/2022<br>08/12/2022 – 09/25/2022	14 days<br>21d ays<br>14 or 7 days with two consecutive RAT negatives<br>3days
Contact quarantined	Close contacts of confirmed cases are required to be quarantined in a quarantine centre or hotel, regardless of their infection status.	01/01/2020 – 02/07/2022<br>02/08/2022 – 12/28/2022	Camp<br>Camp/home
Case isolated	Confirmed cases are required to be isolated at hospital or isolation facility upon testing positive, regardless of their symptoms.	01/23/2020 – 02/07/2022<br>02/08/2022 – 01/30/2023	Mandate hospital isolation<br>Hospital/isolation facility/home
Community-based			
School closure	All kindergartens, primary and secondary schools and private schools in Hong Kong should suspend face-to face classes and all on-campus activities	01/25/2020 – 05/26/2020<br>07/13/2020 – 09/22/2020<br>12/02/2020 - 01/10/2021<br>07/15/2021 – 08/31/2021<br>01/14/2022 – 04/18/2022	Closed
Work-from-home	Special work arrangement for civil servants. Private business was encouraged to follow the work at home arrangements.	01/29/2020 – 03/01/2020<br>03/25/2020 – 05/03/2020<br>07/20/2020 – 08/23/2020<br>11/20/2020 – 01/17/2021<br>01/25/2022 – 04/20/2022	Flexible work, except for essential service
Mask mandate	The mandatory mask-wearing requirement stipulates a person must wear a mask all the time when the person is entering or present in a specified indoor or outdoor public place.	07/15/2020 – 07/28/2020<br>07/28/2020 - 03/01/2023	public indoors<br>public indoors and outdoors

Appendix Table 5. Estimates of the strength of driving factors with backward selection on influenza transmission in the Hong Kong, 2010–2023

Driving Factor	Estimate	95% CI	P value
Between-season intercept	0.830	0.641, 1.087	
Between-season depletion of susceptibles	-0.17	-0.19, -0.118	
Temperature	-0.079	-0.115, -0.044	<0.001
Wind speed	-0.031	-0.059, -0.002	0.035
Mask	-0.318	-0.593, -0.045	0.023
Preventive score 3	-1.446	-2.036, -0.855	<0.001

∗ For regression coefficients that were specific for each season, we estimated the range of R_0S_{0j} to be 0.785-1.024 and the range of z_j to be -1.575 to -0.083 .

Appendix Table 6. Effect of public health and social measures against COVID-19 on time-varying reproduction number of influenza with backward selection, Hong Kong, 2010-2023

PHSM	Description	% Change in R_t (95% CI)
Mask		-27 (-44 to -4)
Preventive score 3	Avoid going to crowded places<br>Avoid going to health care facilities<br>Avoid touching and protect in public	-76 (-87to -57)

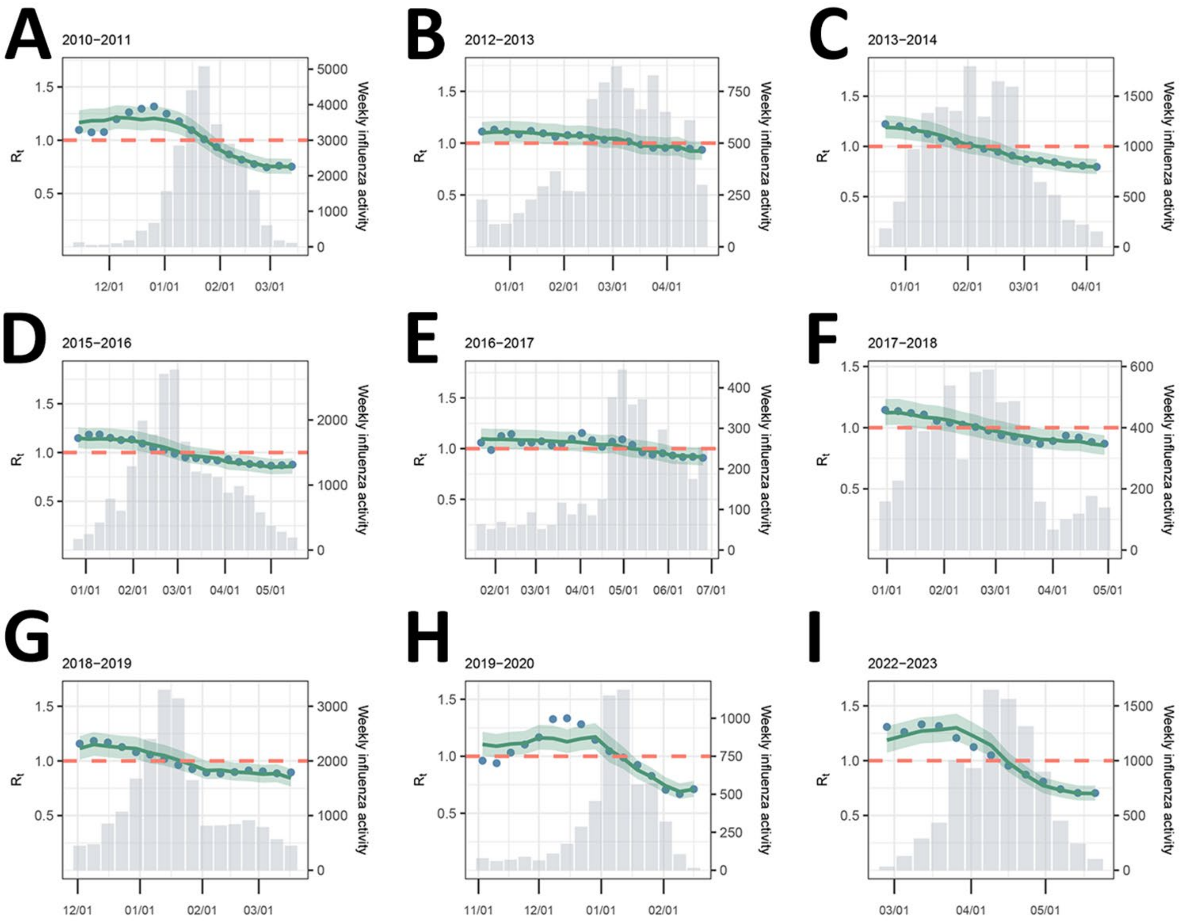

Appendix Figure 1. Influenza reproductive number based on the final model. The grey bar represents the weekly influenza activity in 9 epidemic seasons, showing the peak for each season. The green dot represents the reproduction numbers estimated from the weekly influenza proxy series and the green line represents the predicted reproduction numbers. The shaded area represents the 95% predictive interval.

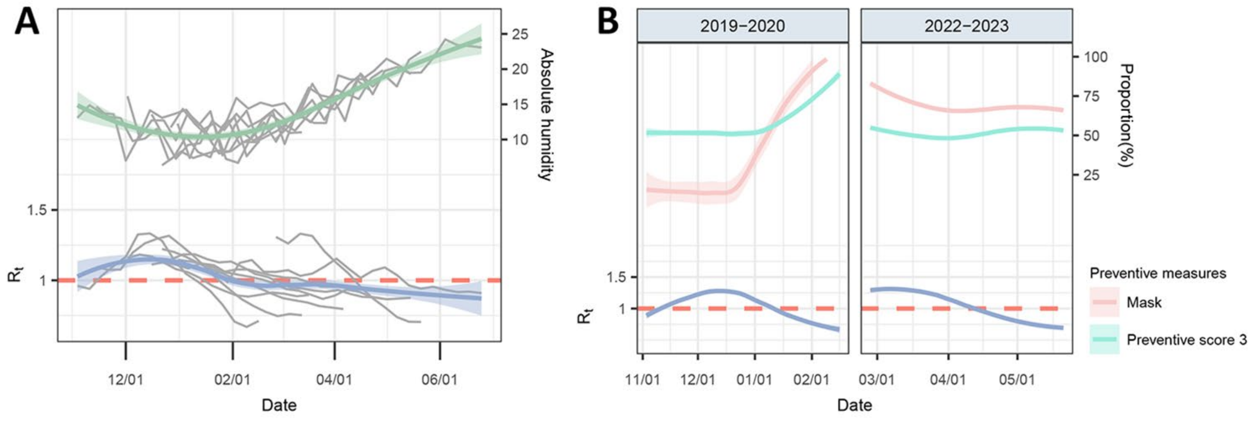

Appendix Figure 2. Estimated reproductive number and driving factors. A) The gray lines represent absolute humidity and reproductive number during each seaso. The green and blue lines represent the smoothed conditional mean of absolute humidity and reproductive number respectively. B) Estimated reproductive number and the proportion of mask wearing and preventive scores during the 2019–20 and 2022–23 influenza seasons.

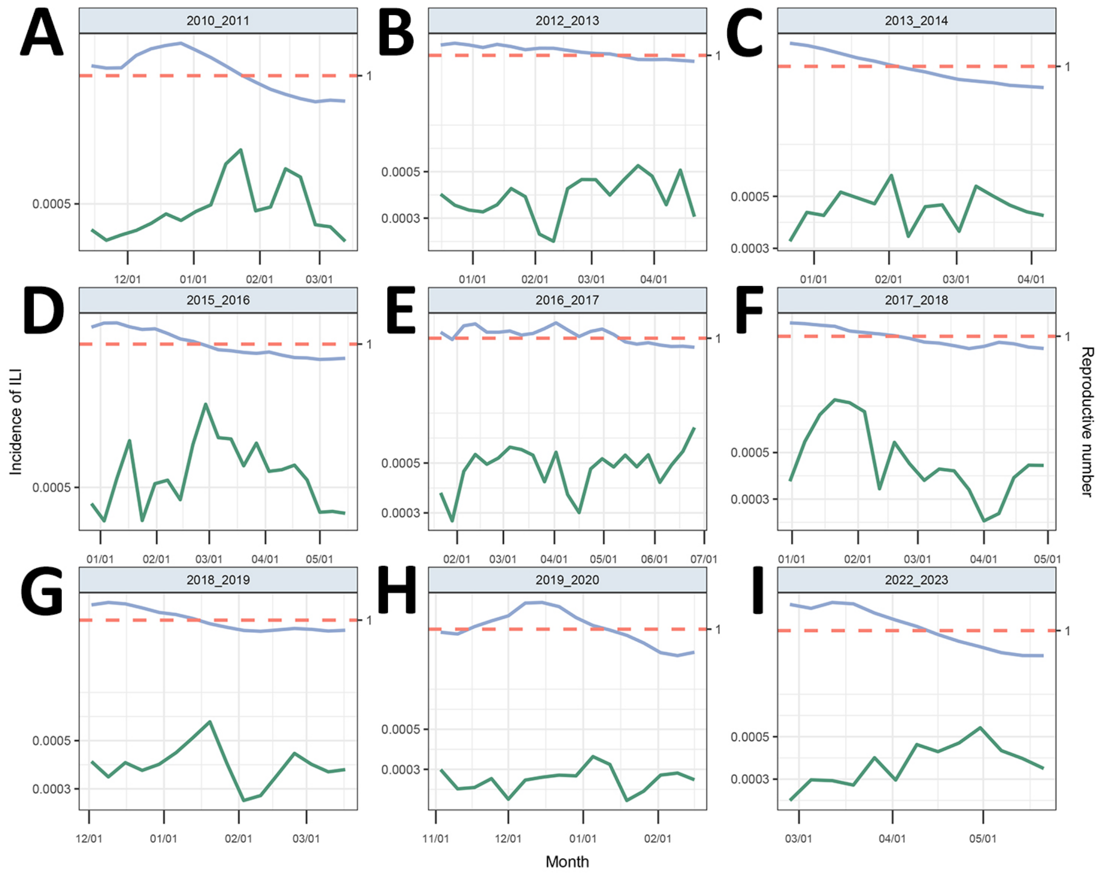

Appendix Figure 3. Time series of incidence of influenza-like-illness (ILI) in Hong Kong over 2010–2023 (bottom) and the reproduction number (top) calculated from the ILI proxy.

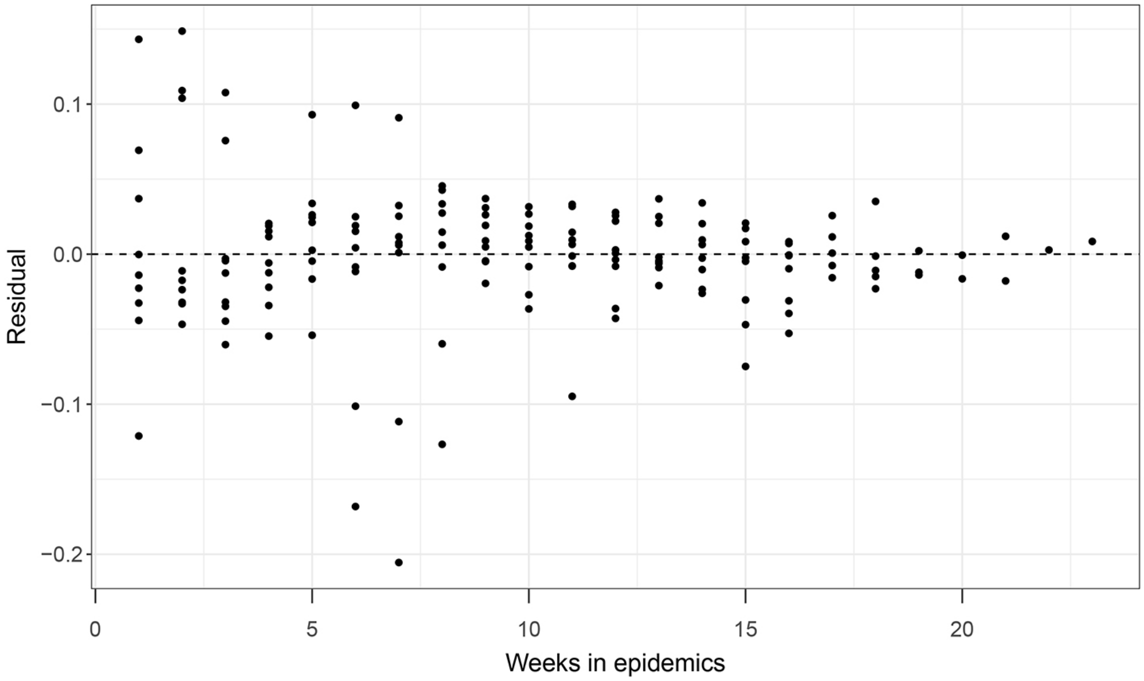

Appendix Figure 4. The residuals of the final model, incorporating depletion of susceptibles over time and between-season effects, absolute humidity, mask and the preventive score, indicating goodness of fit.

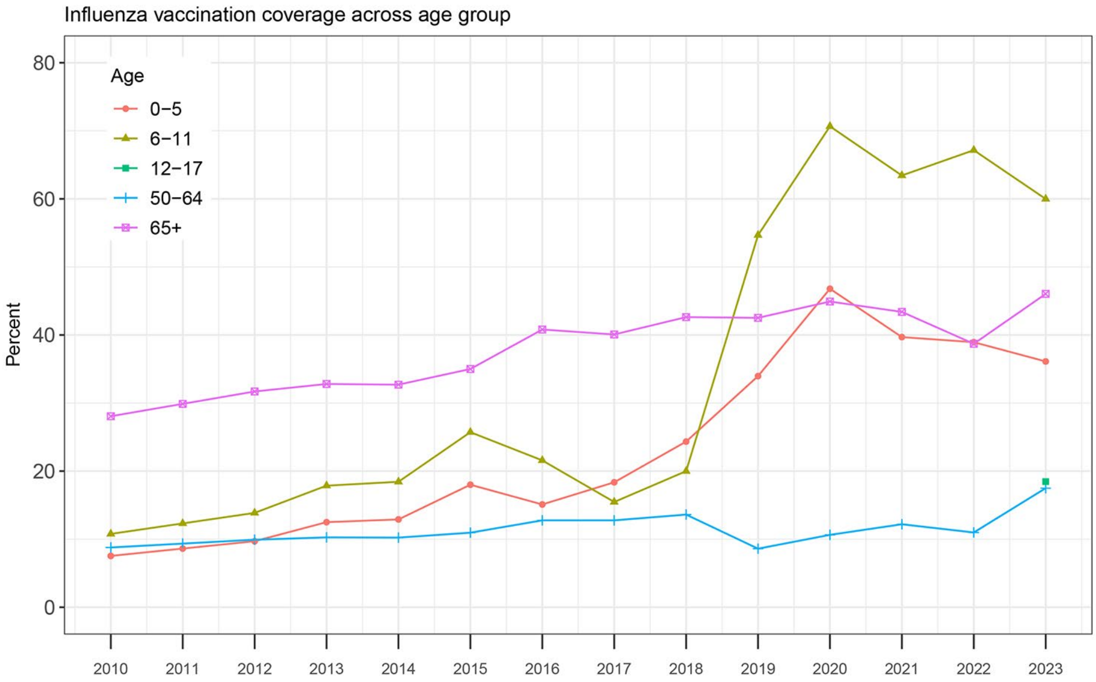

Appendix Figure 5. Pre-season influenza vaccine coverage by age group in Hong Kong during the study period.
